# Immunogenetic Signature of the MICA‐129 Val/Val Genotype Associated With Metastasis in Melanoma

**DOI:** 10.1155/jimr/2883849

**Published:** 2026-11-01

**Authors:** Bruna Somilio da Fonseca, Bruna D. L. Fragelli, Joice M. A. Rodolpho, Krissia F. Godoy, Camila F. B. Castro, Erick C. Casteli, Lucas R. Azevedo, Izabela A. Lopes, Luciana Benevides, Fernanda F. Anibal

**Affiliations:** ^1^ Laboratory of Inflammation and Infectious Diseases Federal University of São Carlos (UFSCar) 13565–908 São Carlos SP Brazil ufscar.br; ^2^ Padre Albino University Centre – UNIFIPA 15809–144 Catanduva Brazil; ^3^ Molecular Genetics and Bioinformatics Laboratory–Experimental Research Unit School of Medicine São Paulo State University (UNESP) Botucatu 18618–687 Botucatu SP Brazil unesp.br; ^4^ Fiocruz Ribeirão Preto Oswaldo Cruz Foundation (Fiocruz) 14049–900 Ribeirão Preto SP Brazil fiocruz.br

**Keywords:** cytokines, genetic polymorphism, melanoma, MHC class I-related chain A (MICA), NKG2D receptor

## Abstract

MICA (major histocompatibility complex class I [MHC class I] chain‐related protein A) is a stress‐induced glycoprotein that functions as a ligand for the NKG2D (natural killer [NK] group 2 member D) receptor, expressed on NK cells and CD8^+^ T lymphocytes (cytotoxic T cells), playing a key role in tumor immune surveillance. In melanoma, modulation of this axis promotes tumor immune evasion. The functional MICA‐129 polymorphism (methionine [Met]/valine [Val]) alters the binding affinity to NKG2D, thereby influencing the cytotoxic immune response. This study included patients with basal cell carcinoma (BCC) and melanoma (primary and metastatic). The Functional Assessment of Cancer Therapy–Melanoma (FACT‐M) questionnaire was administered, followed by MICA‐129 genotyping using quantitative polymerase chain reaction (qPCR), measurement of soluble MICA (sMICA) by enzyme‐linked immunosorbent assay (ELISA), analysis of MICA and NKG2D expression on CD8^+^ T lymphocytes by flow cytometry, and assessment of inflammatory cytokines and angiogenic factors. A higher frequency of the Val/Val genotype was observed in metastatic melanoma, whereas serum sMICA levels did not differ among the groups. The Val variant was associated with reduced functional stability of the MICA–NKG2D axis, favoring tumor immune evasion. Primary melanoma exhibited a more pronounced inflammatory profile, whereas metastatic melanoma showed greater angiogenic activity, indicating a microenvironment conducive to tumor progression. These findings suggest that the MICA‐129 Val/Val genotype may serve as a prognostic biomarker and support the MICA–NKG2D axis as a potential therapeutic target in melanoma.

## 1. Introduction

Melanoma is a malignant neoplasm originating from melanocytes and represents one of the most aggressive forms of skin cancer due to its high metastatic potential, including involvement of the central nervous system (CNS) [1]. The global incidence of melanoma has been progressively increasing, with ~57,000 deaths estimated in 2020 [2]. Five‐year survival rates vary between developed and developing countries, with a global average of 69% [3]. In Brazil, ~8980 new cases are estimated annually between 2023 and 2025, with a predominance in the South and Southeast regions [4].

Melanoma is associated with intrinsic risk factors, such as genetic predisposition and immunosuppression, and extrinsic factors, particularly ultraviolet (UV) radiation exposure [5]. Early diagnosis is an important prognostic factor and is associated with improved clinical outcomes [6]. In this context, genetic studies and the incorporation of molecular markers and genetic panels have shown potential to improve the personalization of melanoma clinical management, although advanced techniques, such as computer‐assisted dermoscopic detection, still face limitations due to lesion variability and the limited availability of labeled images for training artificial intelligence systems [7, 8]˒ From an immunological perspective, natural killer (NK) cells, which belong to the innate immune system, and CD8^+^ T lymphocytes, part of the adaptive immune system, exert cytotoxic functions after recognizing antigens presented by molecules of the major histocompatibility complex class I (MHC class I) [9].

The MHC class I chain‐related protein A (MICA) gene plays a fundamental role in tumor immunosurveillance as its expression increases in tumor cells that exhibit greater chromosomal instability, genotoxic stress, and activation of oncogenic pathways. On the cell surface, MICA acts as a ligand for the NK group 2 member D (NKG2D) receptor, present on NK cells and CD8^+^ T lymphocytes, promoting cytotoxic activation and cytokine production [10, 11].

However, in some tumors, cleavage and release of the soluble form of MICA (soluble MICA [sMICA]) occur. This soluble form interacts with NKG2D without activating it, leading to reduced receptor expression on effector cells, thereby impairing antitumor cytotoxicity and functioning as an efficient mechanism of immune evasion [12, 13].

The functional MICA‐129 dimorphism results in the substitution of methionine (Met) with valine (Val) at position 129, modulating the affinity for the NKG2D receptor and the intensity of activation of NK cells and CD8^+^ T cells. The presence of the Val allele is associated with greater susceptibility to proteolytic cleavage, increasing the levels of sMICA [14] and also influences the density of MICA on the plasma membrane and the release of its soluble form [12].

The melanoma tumor microenvironment is complex and includes various cell types, including stromal cells of the tumor microenvironment [15]; consequently, numerous cytokines are released that can both promote the activation of NK cells and CD8^+^ T cells, as well as limit effector responses and favor immunosuppression [16–20]. In addition, angiogenesis is essential for tumor growth and dissemination and is also regulated by these cytokines [3, 21]. Altogether, this scenario plays a central role in tumor progression and immune evasion.

Although the immunological and angiogenic mechanisms involved in melanoma progression have been extensively studied [22, 23], a significant gap remains regarding the integration of the functional MICA‐129 gene dimorphism into the systematic analysis of immunological and angiogenic markers in this context [24]. To date, the specific biological impact of this polymorphism on the modulation of the MICA–NKG2D pathway and its potential repercussions on tumor immunosurveillance and angiogenesis in melanoma have not been adequately elucidated.

Despite growing evidence supporting the involvement of the MICA–NKG2D axis in tumor immune surveillance, the influence of the functional MICA‐129 polymorphism on the immune cell phenotype and sMICA levels in patients with melanoma remains poorly understood. We hypothesized that the MICA‐129 Val/Val genotype is associated with alterations in the expression of MICA and NKG2D on CD3^+^CD8^+^ T lymphocytes [25], as well as with changes in circulating inflammatory mediators that may contribute to melanoma progression. Therefore, the aim of this study was to investigate the association between the MICA‐129 polymorphism and the immunophenotypic profile of CD3^+^CD8^+^ T lymphocytes, serum sMICA levels, cytokine concentrations, and clinical characteristics in patients with melanoma, evaluating the potential contribution of this immunogenetic axis to the disease progression.

## 2. Methodology

### 2.1. Ethical Aspects of the Research

This study was approved by the Research Ethics Committee of the Federal University of São Carlos (UFSCar) (Protocol Number 6.469.081) and by the Research Ethics Committee of the Padre Albino Foundation (Protocol Number 6.331.159) and is registered in the Brazil Platform, a national unified database for research involving human subjects, part of the Research Ethics Committee/National Research Ethics Commission (CEP/Conep) system. All participants provided written informed consent (ICF) (Supporting Information 1) and were recruited through convenience sampling as volunteers at a private dermatology clinic in the interior of São Paulo state, Brazil.

### 2.2. Participants and Eligibility

Study participants were selected from a review of biopsy reports recorded in the database of the Pathology Laboratory of the Faculdade de Medicina de Catanduva between 2019 and 2021, totaling 17,750 patients. The initial screening was conducted through a retrospective analysis of the available records, aiming to select patients who had not progressed to death. The sample size (*n*) was supplemented with patients from a private clinic in Catanduva, São Paulo. Eligible individuals were included in the study according to pre‐established inclusion and exclusion criteria described as follows:

Inclusion criteria for the basal cell carcinoma (BCC) control group were patients of both sexes, aged over 18 years, with a history of BCC.

Exclusion criteria: individuals under 18 years of age, currently using immunobiological medications, or diagnosed with other malignancies in different organs.

Inclusion criteria for the primary melanoma (M) and metastatic melanoma (M + M) study groups: patients of both sexes, aged over 18 years, with a history of primary melanoma or metastatic melanoma according to the American Joint Committee on Cancer (AJCC) criteria [26].

Exclusion criteria: individuals under 18 years of age, currently using immunobiological medications, or diagnosed with other malignancies in different organs.

Accordingly, the following study groups were established:

• Control group: BCC: *n* = 11.
• Experimental group 1: primary melanoma (M): *n* = 18.
• Experimental group 2: metastatic melanoma (M + M) *n* = 6

During the study period, none of the participants in the three study groups were receiving immunotherapy or corticosteroids nor did they have a history of prior systemic treatments. Only two patients in the M + M group were receiving targeted therapy at the time of study enrollment.

### 2.3. Questionnaire Administration and Sample Collection

After the selection of volunteers, the Functional Assessment of Cancer Therapy–Melanoma (FACT‐M) questionnaire [26] from the Functional Assessment of Chronic Illness Therapy (FACIT) platform (Supporting Information 2) was administered. The questionnaire had been previously translated and adapted for the study population.

The FACT‐M is a quality‐of‐life assessment instrument specifically designed for patients with melanoma, developed from the FACT system created by the FACIT group [27]. It combines the FACT– General (FACT‐G) core, which evaluates general aspects of cancer patients, with a melanoma‐specific module. This module includes the following domains (categories): 1. physical well‐being; 2. social/family well‐being; 3. emotional well‐being; 4. functional well‐being; 5. other concerns, addressing melanoma‐specific issues; 6. surgical procedure, evaluating the site of lesion surgery. Lower scores indicate a worse prognosis.

Subsequently, peripheral blood samples were collected in two 4 mL heparinized tubes and one 8 mL serum tube with gel for subsequent laboratory analyses.

### 2.4. Total Body Mapping for Exclusion of Other Cutaneous Neoplasms

All volunteers in the M + M group underwent total body mapping and digital dermoscopy using the FotoFinder system (FotoFinder Systems GmbH, Germany) to document and evaluate melanocytic lesions across the entire body surface. The examination included the standardized capture of high‐resolution macroscopic and microscopic images of each lesion. Total body mapping was used as a screening tool to exclude the presence of other cutaneous neoplasms in addition to the previously diagnosed melanoma. In the M group, 77% of volunteers underwent total body mapping as the remaining participants had completed mapping at other institutions prior to the study and declined to repeat the procedure.

The BCC group did not undergo total body mapping as participants did not present precursor lesions, and BCC has low metastatic rates. The clinical evaluation and prognostic follow‐up protocol for this group consisted of manual dermoscopy, with no clinical evidence of additional cutaneous neoplasms being observed at the time of assessment.

### 2.5. Immunophenotyping by Multiparametric Flow Cytometry

To evaluate possible alterations in MICA and NKG2D expression on CD8^+^ T lymphocytes, flow cytometry analysis was performed. Peripheral blood mononuclear cells (PBMCs) were isolated from whole blood by density gradient centrifugation using Ficoll after prior dilution of the sample in phosphate‐buffered saline (PBS). Following centrifugation, the mononuclear cell fraction was collected, and the cells were quantified using a Neubauer chamber. Next, 1 × 10^6^ cells were subjected to staining with a viability dye and specific antibodies targeting the markers of interest. The antibodies used in the assay are summarized in Table 1.

**Table 1:** Flow cytometry immunophenotyping panel used for cellular population analysis.

Marker	Fluorochrome	Excitation (nm)	Emission (nm)	Filter
MICA	APC	633–640	~ 660	660/20
CD8	PE	488 or 561	~ 575	575/26
CD3	APC‐Cy7	633–640	~ 785	730/45
NKG2D	PerCP‐Cy.5.5	488 or 561	~ 578	582/15
Viability	BV510	405	~ 510	525/40

*Note:* This table presents the markers evaluated, their conjugated fluorochromes, excitation wavelengths (nm), approximate emission peaks (nm), and optical filters used for detection on the BD FACSymphony A1 Flow Cytometer (BD Biosciences, San Jose, CA, USA).

Sample acquisition was performed using the FACSymphony A1 Flow Cytometer (BD Biosciences, San Jose, CA, USA) at the Flow Cytometry Laboratory of the Fiocruz São Paulo Bi‐Institutional Platform in Ribeirão Preto, with 100,000 events acquired per sample. The representative flow cytometry acquisition and gating strategy are shown in Figure 1.

**Figure 1:**
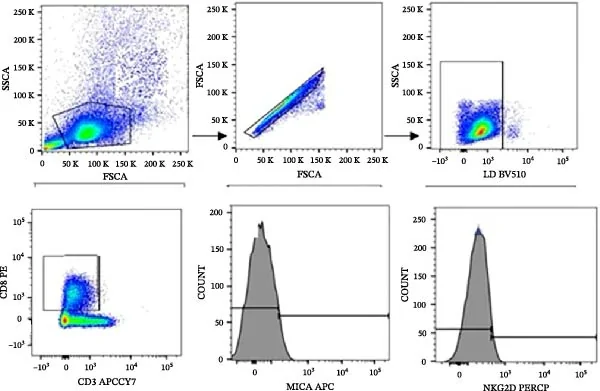
Representative flow cytometry acquisition and gating strategy used for the immunophenotypic analysis of MICA and NKG2D expression in CD3^+^CD8^+^ T lymphocytes. The figure illustrates the sequential acquisition and gating workflow (PBMC selection, doublet exclusion, viability gating, CD3^+^ and CD8^+^ selection) used for data analysis and serves only as a representative demonstration of the acquisition strategy, not as a presentation of experimental results. MICA and NKG2D expression were evaluated exclusively within the viable CD3^+^CD8^+^ T‐cell population. Data were acquired using a FACSymphony A1 Flow Cytometer (BD Biosciences, San Jose, CA, USA) and analyzed with FlowJo v10.7.2.

### 2.6. Quantification of sMICA, Vascular Endothelial Growth Factor (VEGF), and Platelet‐Derived Growth Factor‐BB (PDGF‐BB) by Enzyme‐Linked Immunosorbent Assay (ELISA)

The detection of sMICA was performed using the Human MICA ELISA kit (Thermo Fisher), while VEGF and PDGF‐BB isoform were measured using Quantikine ELISA kits (R&D Systems). Wash buffer and assay buffer were prepared by diluting the concentrated solutions. The human MICA, VEGF, and PDGF‐BB standards were reconstituted and subjected to serial dilutions to generate the standard curve. During the assay, samples and standards were added to the sensitized plate, incubated, and subjected to wash cycles. They were then incubated with secondary antibody + HRP (horseradish peroxidase), followed by reaction with the chromogenic substrate 3,3^′^,5,5^′^‐tetramethylbenzidine (TMB) and stopped with 2 M acidic solution. Optical density was read at 450 nm using a Thermo Scientific Multiskan GO Microplate Spectrophotometer, and concentrations were determined by interpolation from the standard curve. The quantification of sMICA was performed at the Experimental Research Unit (UNIPEX), while the analyses of VEGF and PDGF‐BB were conducted at the Laboratory of Inflammation and Infectious Diseases (LIDI) at the UFSCar.

### 2.7. DNA Extraction and Analysis of the MICA Gene Dimorphism by Quantitative Polymerase Chain Reaction (qPCR)

Genomic DNA was extracted from patient samples using a commercial kit (EasyPure Blood Genomic DNA Kit) and then quantified and diluted to a standardized concentration of 5 ng/µL. The MICA gene dimorphism was analyzed using qPCR with TaqMan probes, employing TaqPath ProAmp Master Mix according to the manufacturer’s protocol. The reaction mix was prepared under sterile conditions and distributed into the qPCR plate, and patient DNA samples were added to reach the final reaction volume. Amplifications were conducted in a StepOnePlus thermocycler in real time using a standardized cycling program. Fluorescence detection allowed the precise identification of the MICA gene dimorphisms. The experiments were performed at the Experimental Research Unit (UNIPEX).

### 2.8. Cytokine Analysis by Chemiluminescent Assay

The concentrations of the cytokines interleukin‐1 beta (IL‐1β), tumor necrosis factor alpha (TNF‐α), interleukin‐6 (IL‐6), and interleukin‐8 (IL‐8) were determined by automated chemiluminescent immunoassay using the Immulite 1000 system (Siemens Healthcare Diagnostics) according to the manufacturer’s instructions. Samples were thawed, homogenized, and processed automatically by an instrument. Cytokines in the samples bound to specific immobilized antibodies, followed by the addition of a chemiluminescent‐labeled secondary antibody. All assay steps were performed automatically by the equipment. A signal proportional to cytokine concentration was generated and quantified by comparison with reference values provided by the manufacturer (IL‐6 = 7 pg/mL; IL‐8 = 15 pg/mL; IL‐1β = 5 pg/mL; and TNF‐α = 8 pg/mL). The results were expressed in pg/mL. The analyses were performed at the LIDI of the UFSCar.

### 2.9. Statistical Analysis

The data obtained in this study were analyzed using GraphPad Prism software, version 9.0 (San Diego, California, USA). Outliers were identified using the Grubbs test, followed by the Shapiro–Wilk test to assess data normality.

For parametric data, analysis of variance (ANOVA) was applied, followed by Tukey’s multiple comparison test, and results were expressed as the mean ± standard deviation (SD). For nonparametric data, the Kruskal–Wallis test was used, followed by Dunn’s multiple comparison test, with results expressed as the median and interquartile range [Me (Q1; Q3)]. A *p*‐value ≤ 0.05 was considered statistically significant.

## 3. Results

### 3.1. Clinical Questionnaire Results

In Figure 2, the scores obtained from the FACT‐M questionnaire are tabulated. In Figure 2A, corresponding to the physical score domain, a significant difference was observed, with the M + M group showing a lower score compared to both the BCC and M groups, indicating a worse quality of life in this domain. In Figure 2E,G, corresponding to the other concerns and total score domains, which relate to systemic symptoms and the functional impact of the disease, a significant difference was observed in the M + M group, which presented lower scores compared to the other groups.

**Figure 2:**
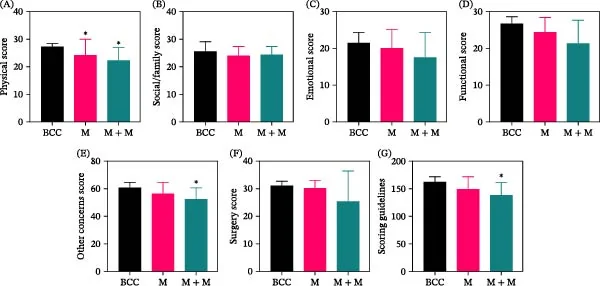
Comparison of FACT questionnaire scores among the groups basal cell carcinoma (BCC), melanoma (M), and metastatic melanoma (M + M). (A) Physical parameters. (B) Social and family parameters. (C) Emotional parameters. (D) Functional parameters. (E) Other concerns. (F) Surgical parameters. (G) Total score. ( ^∗^) vs. BCC:  ^∗^ *p* ≤ 0.05. Results are presented as median with lower and upper quartiles: Me (Q1; Q3). Statistical analysis was performed using the nonparametric Kruskal–Wallis test.

### 3.2. Total Body Skin Mapping

In the total body skin mapping, three patients in the M group presented additional cutaneous neoplasms: two patients were diagnosed with BCC and the third had a second melanoma. In the M + M group, three patients also presented additional neoplasms: two were diagnosed with BCC, and the third had a second melanoma, in addition to 8 lesions suggestive of BCC. These findings did not lead to the exclusion of any participants according to the pre‐established exclusion criteria.

### 3.3. Biomarker Analysis

Table 2 shows the distribution of MICA‐129 dimorphism genotypes (Val and Met) among the evaluated groups. In the BCC group, the Met/Val genotype predominated, present in 9 of 11 individuals, while the Met/Met genotype was identified in 2 individuals, and the Val/Val genotype was absent. In the M group, the Met/Val genotype was most frequent, detected in 11 of 17 individuals, followed by the Val/Val genotype in six individuals, with no Met/Met genotype observed. In the M + M group, only homozygous genotypes were observed: four individuals with Val/Val and two with Met/Met, with no heterozygous Met/Val genotype detected. Considering the total sample (*n* = 34), the Met/Val genotype was the most frequent, followed by Val/Val and Met/Met. A detailed table with alleles for each sample can be found in Table S1.

**Table 2:** Distribution of MICA‐129 genotypes (Met/Met, Val/Val, and Met/Val) among the studied groups.

Group	Met/Met	Val/Val	Met/Val	Total
BCC	2	0	9	11
M	0	6	11	17
M + M	2	4	0	6
Total	4	10	20	34

*Note:* Absolute distribution of MICA‐129 polymorphism genotypes in the groups basal cell carcinoma (BCC), melanoma (M), and metastatic melanoma (M + M), as well as in the total sample (*n* = 34).

Abbreviations: Met, methionine; Val, valine.

In Figure 3, the quantifications of sMICA and the determination of the MICA‐129 dimorphism are presented. In Figure 3A, serum sMICA levels showed substantial interindividual variability across the three groups. A considerable overlap was observed among the BCC, M, and M + M groups, with no clear visual separation of the medians. Overall, the observed pattern suggests no significant difference among the groups with respect to circulating sMICA levels.

**Figure 3:**
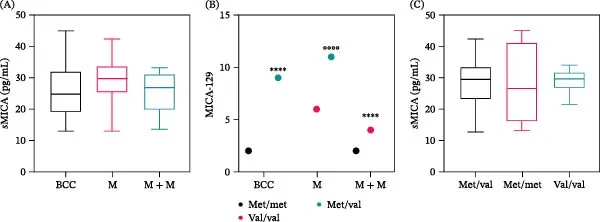
Quantification of soluble MICA and determination of MICA‐129 dimorphism in basal cell carcinoma (BCC), melanoma (M), and metastatic melanoma (M + M) groups. (A) Serum levels of soluble MICA measured by ELISA. (B) Genotypic identification of MICA‐129 dimorphisms by qPCR. ( ^∗^) vs. Met/Met:  ^∗∗∗∗^ *p* ≤ 0.0001. (°) vs. Val/Val: °°°°*p* ≤ 0.0001. (C) Serum levels of soluble MICA (sMICA) according to MICA‐129 genotype (Met/Val, Met/Met, and Val/Val). Results are presented as median with lower and upper quartiles: Me (Q1; Q3). Statistical analysis was performed using the nonparametric Kruskal–Wallis test.

In Figure 3B, the genotypic distribution (Met/Met, Val/Val, and Met/Val) across the three groups is presented. In the BCC group, the Met/Met genotype predominated and was associated with significantly lower values, whereas the Met/Val genotype exhibited higher levels, and the Val/Val genotype was not represented graphically. In the M group, a significant difference was observed for the Met/Val genotype, whereas the Met/Met genotype was not detected. In the M + M group, the homozygous Val/Val and Met/Met genotypes predominated, with a significant increase in Val/Val, while the Met/Val genotype was not detected.

In Figure 3C, serum sMICA levels are presented according to the MICA‐129 genotype. Substantial variability in sMICA levels was observed among individuals, regardless of the genotype. The median values were similar among the Met/Val, Met/Met, and Val/Val genotypes. Therefore, no evident difference in serum sMICA levels was observed among the different MICA‐129 genotypes.

Figure 4 presents the frequency of NKG2D and MICA expression in CD8^+^ T lymphocytes. As shown in Figure 4A, cell surface MICA expression did not differ significantly among the analyzed groups. However, a greater dispersion of values was observed in the M + M group compared with the M and BCC groups. Similarly, the analysis of NKG2D expression (Figure 4B) revealed no statistically significant differences among the groups, although the M and M + M groups showed similar median values that were higher than those observed in the BCC group.

**Figure 4:**
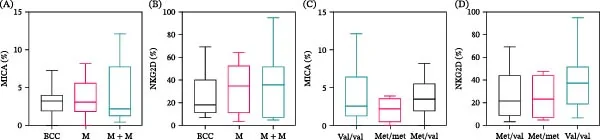
Cellular expression profile of MICA and NKG2D (%) on CD8^+^ T lymphocytes. (A) Percentage of CD8^+^ T lymphocytes expressing MICA in the BCC, M, and M + M groups. (B) Percentage of CD8^+^ T lymphocytes expressing NKG2D in the BCC, M, and M + M groups. (C) Percentage of CD8^+^ T lymphocytes expressing MICA according to the MICA‐129 genotype (Val/Val, Met/Met, and Met/Val). (D) Percentage of CD8^+^ T lymphocytes expressing NKG2D according to the MICA‐129 genotype (Met/Val, Met/Met, and Val/Val). Results are presented as median with lower and upper quartiles: Me (Q1; Q3). Statistical analysis was performed using the nonparametric Kruskal–Wallis test.

In the analysis according to genotype (Figure 4C), no significant differences in MICA expression were observed among the Val/Val, Met/Met, and Met/Val genotypes. The Val/Val group showed a wider distribution range, whereas the Met/Met and Met/Val groups exhibited more concentrated values. For NKG2D (Figure 4D), no statistically significant differences were identified among the genotypes. The Val/Val group showed the highest median and the widest distribution range, whereas the Met/Met group showed the lowest median values and the Met/Val group showed intermediate values.

Figure 5 presents the inflammatory profile of the analyzed patients. In Figure 5A, a significant increase in the inflammatory cytokine IL‐6 was observed in the M group compared to that in the BCC group. In the M + M group, values were intermediate, lower than those in the M group, and closer to the BCC group. In Figure 5B, IL‐8 levels were elevated in the M group compared to those in the BCC, while the M + M group showed values similar to those in the M group. In Figure 5C, a progressive increase in TNF‐α was observed across the groups: the BCC group had the lowest levels, the M group showed moderate elevation, and the M + M group exhibited the highest values. In Figure 5D, IL‐1β levels remained essentially similar among the three groups, with no relevant differences observed. In Figure 5E, VEGF levels in the BCC group showed wide variability with elevated values. The M group exhibited a general reduction compared to BCC, while the M + M group showed an increase compared to M, with levels closer to those of the BCC group. In Figure 5F, the highest PDGF‐BB levels were observed in the BCC group. The M group had reduced values, and the M + M group maintained low levels similar to those of the M group.

**Figure 5:**
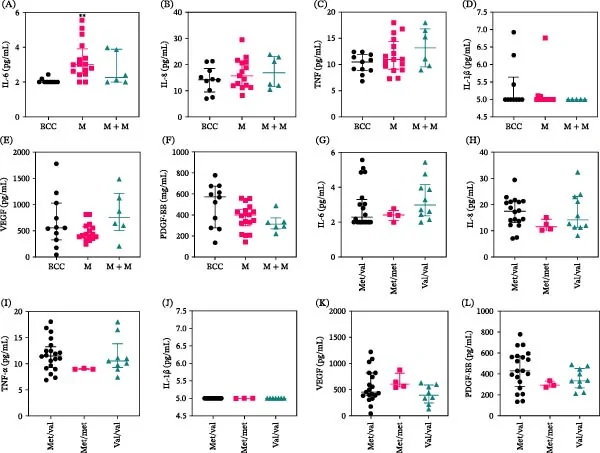
Serum levels of cytokines and growth factors in the basal cell carcinoma (BCC), melanoma (M), and metastatic melanoma (M + M) groups, and according to the MICA‐129 genotype. (A) Serum IL‐6 levels in the BCC, M, and M + M groups. (B) Serum IL‐8 levels in the BCC, M, and M + M groups. (C) Serum TNF‐α levels in the BCC, M, and M + M groups. (D) Serum IL‐1β levels in the BCC, M, and M + M groups. (E) Serum VEGF levels in the BCC, M, and M + M groups. (F) Serum PDGF‐BB levels in the BCC, M, and M + M groups. Serum levels according to the MICA‐129 genotype (Met/Val, Met/Met, and Val/Val): (G) IL‐6; (H) IL‐8; (I) TNF‐α; (J) IL‐1β; (K) VEGF; and (L) PDGF‐BB. ( ^∗^) vs. BCC:  ^∗∗^ *p* ≤ 0.01. Results are presented as the median with lower and upper quartiles: Me (Q1; Q3). Statistical analysis was performed using the nonparametric Kruskal–Wallis test.

In Figure 5G, IL‐6 levels vary according to the genotype. The Met/Val group showed relatively lower and more homogeneous values, whereas the Val/Val group exhibited higher levels and greater variability. The Met/Met group showed intermediate values. In Figure 5H, IL‐8 levels were higher and showed greater variability in the Met/Val group, whereas the Met/Met group exhibited lower values. The Val/Val group showed intermediate to high IL‐8 levels with considerable variability. In Figure 5I, TNF‐α levels showed greater variability in the Met/Val group, including some individuals with elevated concentrations. The Met/Met group showed relatively lower and more homogeneous values, whereas the Val/Val group exhibited intermediate levels.

In Figure 5J, IL‐13 levels remained essentially constant across the three genotypes, with values close to 5 pg/mL and no apparent differences among the groups.

In Figure 5K, VEGF concentrations showed wide variability in the Met/Val group, including individuals with elevated values. The Met/Met group exhibited relatively higher concentrations than the Val/Val group, whereas the latter showed lower and more homogeneous values. In Figure 5L, PDGF‐BB levels showed high variability in the Met/Val group, with some individuals exhibiting elevated concentrations. The Met/Met group showed lower and more homogeneous values, whereas the Val/Val group exhibited intermediate concentrations and moderate variability.

## 4. Discussion

The FACT‐M questionnaire was adapted for the Brazilian population, allowing the inclusion of the BCC group, and the melanoma‐specific concerns were replaced with additional concerns, encompassing patients with both carcinoma and melanoma. Studies on reliability, validity, and responsiveness have demonstrated that FACT‐M is a robust and widely used instrument in various clinical contexts [28].

The results showed significant differences between the study groups. In the physical well‐being domain, the additional concerns domain, and the total FACT‐M score, the M + M group had the lowest scores, reflecting a greater disease impact and indicating a globally impaired quality of life in more advanced stages. A previous study demonstrated that patients with more advanced melanoma stages had worse scores in the same domains, supporting the notion that disease progression negatively affects the overall quality of life [29].

It was observed, however, that the emotional well‐being, social/family, and functional domains showed greater heterogeneity between studies, with scores that in some cases remained close to normative averages, depending on the population assessed and cultural context [30]. Overall, the data indicate that although disease progression is generally associated with a decline in quality of life, the magnitude of this impact can vary according to clinical and contextual characteristics, reflecting the differences observed in populations with distinct clinical and therapeutic profiles. FACT‐M is sensitive to these variations, even when applied to severe patients with other cutaneous neoplasms [31].

Additionally, patients with 10 or more tumors had significantly lower total FACT‐M scores, as well as lower scores in the emotional well‐being and functional domains and in the melanoma‐specific subscale, which corresponds to additional concerns in the adapted version [29]. These results reinforce the relationship between greater clinical severity and poorer quality of life, demonstrating that FACT‐M is sensitive to detecting significant differences between subgroups of patients with distinct clinical characteristics [32].

Thus, melanoma progression impacts not only biological parameters but also the patient’s overall physical condition and functionality [28, 33].

In addition to the clinical and functional impacts of melanoma, biological studies have investigated molecular factors that modulate disease progression [34]. Among these factors, the MICA‐129 dimorphism has been extensively studied in solid tumors due to its functional effect on the expression of stress‐induced MICA molecules and on the activation of immune cells through the NKG2D receptor, as discussed in recent reviews [13, 35, 36].

Considering the functional role of the MICA/NKG2D axis in tumor immunosurveillance, factors present within the tumor microenvironment may interfere with the activation of effector cells. Immunosuppressive cytokines, such as TGF‐β, can negatively regulate NK cell‐mediated antitumor responses. Previous studies have demonstrated that elevated TGF‐β levels are associated with reduced NKG2D expression and impaired cytotoxic activity of NK cells in melanoma patients, suggesting an additional mechanism of tumor immune evasion [37]. Similarly, the release of soluble NKG2D ligands, such as sMICA, may negatively modulate the activation of NK cells and CD8^+^ T lymphocytes in solid tumors, including melanoma, contributing to immune escape mechanisms and compromising the effectiveness of antitumor responses [38]. In the sample of this study, the M + M group, considered clinically more severe, showed a predominance of Val/Val (four/six individuals), with Met/Met in two individuals and no Met/Val detected. In the BCC and M groups, Met/Val was predominate. A previous clinical study evaluated the frequency of MICA‐129 genotypes in patients with cutaneous melanoma compared to healthy controls but did not observe statistically significant differences between the groups nor a consistent association with clinical features such as tumor thickness or ulceration, indicating that isolated variation may not determine disease risk or severity [39].

In the present study, it was observed that circulating sMICA levels and the cell‐surface expression of MICA and NKG2D on CD8^+^ T lymphocytes did not show statistically significant differences among the analyzed groups.

The absence of significant differences in circulating sMICA levels among the groups may be explained by several factors. The small sample size may have limited the statistical power to detect differences, while the high interindividual variability in serum sMICA levels, influenced by clinical and biological factors, may have contributed to the dispersion of the results [40]. Furthermore, MICA expression and shedding may be regulated predominantly within the tumor microenvironment and may not necessarily be reflected in detectable changes in the systemic circulation. Another aspect to be considered is that two patients in the M + M group were receiving targeted therapy, which may have influenced sMICA expression and shedding [41]. Finally, differences in tumor burden, including the number of lesions, tumor size, and the location of metastases, may also have affected circulating sMICA concentrations [42].

Additionally, in our flow cytometry analysis, the MICA expression was detected in the CD3^+^CD8^+^ T cell population. Although MICA is classically described as a stress‐induced ligand, predominantly expressed by tumor cells, the presence of this molecule on CD8^+^ T lymphocytes can be interpreted under two main hypotheses [43, 44].

First, in the tumor microenvironment of melanoma, there is intense immune activation, in which infiltrating CD8^+^ T lymphocytes (TILs) may experience metabolic stress, chronic activation, continuous exposure to reactive oxygen species (ROS), and a persistent inflammatory environment [45, 46]. Evidence in the literature indicates that immune cells under these conditions are capable of modulating the expression of molecules associated with stress and cellular damage pathways, which could explain the endogenous expression of MICA [11, 47, 48].

Alternatively, the detected signal may reflect membrane transfer events, such as trogocytosis, during tumor‐immune interactions. CD8^+^ T lymphocytes are capable of acquiring membrane fragments from tumor cells after immunological contact, a phenomenon widely described in solid tumors, which could explain the detection of MICA on these cells [49–51]. However, due to the absence of specific tumor markers in the panel used, it is not possible to conclusively determine whether the observed signal corresponds to endogenous MICA expression or the presence of acquired membrane fragments [52].

Although endogenous expression and trogocytosis are possible mechanisms to explain the presence of MICA on CD8^+^ T lymphocytes, other possibilities should also be considered as trogocytosis can promote the acquisition of surface molecules derived from other cells [52]. The detection of this molecule may reflect not only expression by the cell itself but also the acquisition of MICA present in the tumor microenvironment, including the binding of sMICA to the surface of lymphocytes, a mechanism described as a potential modulator of the antitumor immune response [53].

Furthermore, the transfer of molecules associated with extracellular vesicles, which are important mediators of intercellular communication, as well as potential technical limitations related to flow cytometry detection, should be considered [54, 55]. The absence of evaluation of activation markers, such as CD69, HLA‐DR, and CD25, represents a limitation of the study as these markers are used to characterize the activation status and functional profile of T lymphocytes, aiding in the interpretation of phenotypic alterations observed by flow cytometry [56, 57].

In addition to the influence of the MICA‐129 polymorphism, the generation of sMICA is strongly regulated by posttranslational mechanisms. Proteolytic shedding of membrane‐bound MICA is mediated by members of the ADAM (a disintegrin and metalloproteinase) family, primarily ADAM10 and, to a lesser extent, ADAM17, reducing the availability of MICA on the tumor cell surface while increasing the circulating levels of sMICA. In turn, sMICA can induce the internalization and downregulation of the NKG2D receptor on NK cells and cytotoxic T lymphocytes, thereby impairing their antitumor activity and promoting immune evasion. Recent studies in melanoma have demonstrated that ADAM10 plays a central role in this process, as its pharmacological or genetic inhibition reduces sMICA release, preserves membrane‐bound MICA expression, and restores NKG2D‐dependent NK cell cytotoxicity [58, 59].

Furthermore, it has been demonstrated that androgen receptor activation can induce ADAM10‐dependent MICA shedding in melanoma cells, establishing an additional mechanism by which tumors regulate the MICA/NKG2D axis and reduce NK cell‐mediated immune surveillance [60].

In in vitro studies using solid tumor cell lines, Val/Val was associated with higher MICA expression on the plasma membrane and increased sMICA release [12], whereas Met/Met showed higher binding affinity to NKG2D, promoting robust activation of NK and CD8^+^ T cells, and the heterozygous Met/Val exhibited an intermediate effect [14].

Furthermore, NKG2D signaling in hematopoietic stem cells can be modulated by the MICA‐129 dimorphism, affecting cell function and survival and demonstrating that these variants regulate immune response efficacy in a context‐dependent manner [14, 61]. These findings indicate that the dimorphism influences MICA expression and shedding, modulating the antitumor immune response and promoting potential immune escape mechanisms, although the data are derived from experimental models rather than clinical melanoma.

Evidence from studies in autoimmune diseases indicates that the MICA‐129 dimorphism modulates the activation of NK cells and CD8^+^ T lymphocytes via the NKG2D receptor [62]. Together with data associating the MICA‐129 Val/Val genotype with an increased risk of nasopharyngeal carcinoma, these findings suggest that the functional effects of the dimorphism, particularly the Val variant, may influence susceptibility to different types of diseases and tumors [63].

In a functional study conducted in hematopoietic stem cell transplantation, it was demonstrated that the MICA‐129 dimorphism, including the Val/Val genotype, exerts a complex effect on NKG2D signaling, with implications for the activation and regulation of these cells [14].

The Met variant exhibits higher affinity for the receptor, promoting stronger signaling and greater initial activation of NK and CD8^+^ T cells, with increased cytotoxicity and interferon‐γ (IFN‐γ) release compared to the Val isoform. However, this robust activation is followed by a faster and more pronounced downregulation of NKG2D on the cell surface, indicating that the MICA‐mediated immune response depends not only on the amount of soluble ligand but also on the specific genotype and can further be modulated by individual patient characteristics, the tumor microenvironment, and concomitant therapies [14, 36].

In light of these data, patients in the M + M group may exhibit higher MICA expression and increased sMICA release, potentially resulting in inhibition of NK and CD8^+^ T‐cell activity and promoting immune escape, whereas in the BCC and M groups, the predominance of Met/Val suggests an intermediate immune activity, balancing MICA expression and effector cell activation.

A study in multiple myeloma highlighted the functional role of sMICA in modulating the immune response and disease progression. It showed that MICA is induced in malignant plasma cells by DNA damage signals and that patients with multiple myeloma exhibit elevated sMICA levels, which can suppress NKG2D receptor expression on NK and cytotoxic T cells, reducing antitumor cytotoxicity and promoting disease progression [64]. Complementing these findings, it was demonstrated that the MICA‐129 dimorphism influences sMICA levels and clinical outcomes in multiple myeloma, with the Val/Val genotype associated with higher serum sMICA concentrations and an increased risk of disease progression [65]. Together, these studies indicate that both sMICA levels and the MICA‐129 genetic variant play critical roles in immunoregulation and prognosis in multiple myeloma, emphasizing the importance of considering phenotypic and genotypic factors in the assessment of tumor progression [64, 65].

Similarly, a clinical study in melanoma showed that elevated sMICA levels in a patient with metastatic or advanced melanoma were associated with reduced response to immunotherapy and poorer clinical outcome, suggesting that increased sMICA may contribute to tumor immune evasion by negatively modulating the activation of NK cells and CD8^+^ T cells via the NKG2D receptor [66].

These findings reinforce that although previous studies in melanoma and multiple myeloma suggest clear effects of sMICA and the MICA‐129 polymorphism, in the human population, the results can be modulated by multiple individual factors, including immunological, genetic, and tumor microenvironment characteristics [64, 65]. Thus, the regulation of MICA and NKG2D constitutes a multifactorial and complex process, which goes beyond interpretation based solely on serum sMICA levels or the presence of a specific genotype [13, 67].

In addition to factors related to MICA and NKG2D, other tumor microenvironment mediators, such as proinflammatory cytokines, play a central role in regulating immunity and melanoma progression [68]. Among these, IL‐6 has been shown to be a crucial modulator, capable of influencing both chronic inflammation and immunosuppression, contributing to therapy resistance and tumor adaptation to the microenvironment [69]. In the present study, the M group exhibited a significant increase in the inflammatory cytokine IL‐6, whereas in the M + M group, the values were intermediately lower than those in the M group and closer to those observed in the BCC group.

IL‐6 contributes to resistance to various oncological treatment modalities, acting not only as an inflammatory cytokine but also as a central communication hub between the tumor and the tumor microenvironment. It promotes chronic inflammation, immune evasion, cellular plasticity, and survival under therapeutic stress [70].

Evidence from BCC indicates that IL‐6 promotes the formation of an immunosuppressive and permissive inflammatory microenvironment, contributing to tumor cell survival and progression [71, 72]. Similarly, in melanoma patients, elevated IL‐6 levels have been associated with resistance to immunotherapy, increased risk of disease progression, shorter progression‐free survival, and poorer clinical outcomes [73, 74]. Experimental studies demonstrate that IL‐6 can be produced autocrinely by tumor cells, forming an inflammatory loop regulated by the transcription factor E2F1, which alters the T‐cell phenotype, promoting immunosuppression and resistance to immunotherapy [75].

In murine melanoma models, IL‐6 increased the immunosuppressive capacity of the tumor microenvironment and inhibited CD8^+^ T lymphocytes; however, isolated IL‐6 blockade was ineffective in vivo and, in some cases, accelerated tumor progression, highlighting the complex and context‐dependent effects of this cytokine [76].

Furthermore, local IL‐6 production by fibroblasts and stromal cells promotes protumor mechanisms, including cell survival, migration, invasion, and angiogenesis, contributing to therapeutic resistance and tumor progression in metastatic melanoma patients [17, 30, 74]. These findings suggest that IL‐6 may play a relevant role in the early modulation of the tumor microenvironment, as observed by its increased levels in primary melanoma, whereas its regulation in metastatic stages may be influenced by the complexity of the tumor microenvironment and by adaptive mechanisms.

In the present study, IL‐8 levels were higher in the M and M + M groups compared to BCC, suggesting that this cytokine plays a relevant role both in early tumor progression and in maintaining the protumoral microenvironment in melanoma. IL‐8 acts as a promoter of angiogenesis, recruitment of immunosuppressive cells, and invasive phenotype, supporting tumor vascularization, nutritional supply, and metastatic dissemination, partly through the regulation of VEGF, and also contributes to resistance to immunotherapy [77].

In the context of melanoma, IL‐8 also functions as a proangiogenic mediator, stimulating new blood vessel formation, endothelial cell proliferation, and tumor stroma remodeling, in conjunction with VEGF and PDGF, which are secreted by both tumor cells and the tumor microenvironment [78].

Thus, IL‐8, VEGF, and PDGF play central roles in angiogenesis and metastatic progression of human melanoma, promoting endothelial proliferation and migration as well as vascular remodeling [79]. Moreover, in patients with advanced melanoma, circulating levels of IL‐8 serve as a negative prognostic biomarker, being associated with a poorer treatment response and reduced survival, reflecting its role in modulating the tumor microenvironment and disease progression [80].

In melanoma, TNF‐α stimulates the production of IL‐6 and IL‐8, creating a proinflammatory tumor microenvironment that promotes cell survival, chronic inflammation, migration, invasion, and angiogenesis, thereby enhancing tumor progression and therapeutic resistance [81]. These protumor effects are reflected in the present study, which showed a progressive increase in TNF‐α levels across the study groups: lowest in BCC, moderate in M, and highest in M + M, suggesting an association with tumor progression and advanced disease stage.

Moreover, TNF‐α promotes cellular differentiation, endowing tumor cells with a more invasive phenotype and enhanced immune evasion capacity, while metabolic changes associated with this cytokine correlate with resistance to immunotherapy, reinforcing its role in tumor aggressiveness [82]. Complementing these findings, a clinical study demonstrated that elevated plasma TNF‐α levels and activation of CD8^+^ T lymphocytes are associated with poorer responses to immune checkpoint inhibitors, indicating that persistent TNF‐α elevations during treatment can compromise the efficacy of immunotherapy [83].

Unlike other proinflammatory cytokines such as TNF‐α, IL‐6, and IL‐8, the endogenous production of IL‐1β by melanoma tumor cells is limited, with the majority of this cytokine being derived from macrophages and other cells in the tumor microenvironment [84]. In the present study, IL‐1β levels remained virtually unchanged across the three groups, reinforcing its stability in the basal homeostasis of melanoma.

A translational study demonstrated that IL‐1β expression is even lower in uveal melanoma compared to that in cutaneous melanoma, suggesting that under homeostatic conditions, this cytokine does not contribute significantly to tumor inflammation or spontaneous tumor progression [85].

When activated, IL‐1β can induce the production of angiogenic factors, such as VEGF, promoting tumor microenvironment remodeling and angiogenesis and potentially enhancing disease progression [86]. Furthermore, studies show that macrophage activation in the tumor microenvironment, associated with the production of soluble mediators, can contribute to tumor growth and disease progression, highlighting the role of interactions between immune cells and tumor cells in this context [87].

In the observed results, the BCC group exhibited high levels of VEGF, indicating active basal angiogenesis in the cutaneous tumor tissue. This finding is consistent with a clinical study that evaluated VEGF expression in BCC samples by immunohistochemistry and found a significant correlation between elevated VEGF levels and features of more aggressive tumors, such as greater invasion depth and larger size, suggesting its potential as a clinical marker to predict aggressiveness and guide therapeutic management [88].

In the M group, VEGF levels were lower, reflecting an early stage of the disease with reduced angiogenic stimulus, in agreement with the reviews on the role of VEGF in tumor progression [89, 90]. Furthermore, melanoma can exploit alternative blood supply mechanisms, such as vasculogenic mimicry, promoting tumor progression, metastasis, and therapeutic resistance [91].

In the M + M group, an increase in VEGF expression was observed, highlighting the reactivation of angiogenesis associated with tumor progression and greater aggressiveness. In preclinical melanoma models, active immunotherapy targeting VEGF reduced tumor growth, suppressed angiogenesis, and increased T‐cell infiltration into the tumor microenvironment, suggesting that strategies combining angiogenesis blockade with immune modulation may be promising in VEGF‐dependent tumors [92]. The findings of the present study reinforce VEGF as a marker of tumor progression and a potential therapeutic target, as described in reviews highlighting VEGF overexpression in advanced and metastatic melanomas [90].

Similar to IL‐8, both VEGF and PDGF are proangiogenic factors that play crucial roles in melanoma progression. Studies indicate that in primary melanoma, the expression of these factors tends to be moderate, reflecting initial and limited angiogenesis. In advanced and metastatic stages, there is an upregulation of VEGF and PDGF, associated with reactivation of angiogenesis, increased tumor vascularization, and enhanced invasive potential, suggesting that regulation of these proangiogenic factors directly contributes to tumor aggressiveness [79, 90]. This phenomenon is also linked to melanoma’s ability to modulate fibroblasts and induce a protumoral phenotype in the tumor microenvironment [93].

In the present study, the highest levels of PDGF‐BB were observed in the BCC group, indicating active basal angiogenesis in BCC. In the M group, PDGF‐BB levels were reduced, suggesting an early stage with limited angiogenesis [94, 95]. PDGF/PDGFR signaling is widely involved in carcinogenesis, tumor progression, invasion, and metastasis across various cancer types, functioning as an important regulator of tumor growth and dissemination [96].

In the M + M group, PDGF‐BB levels remained low, similar to those observed in the M group, indicating that despite tumor progression, the contribution of PDGF‐BB may be lower in metastatic stages, unlike VEGF, which is reactivated and becomes more relevant in advanced tumor angiogenesis. This highlights the differential roles of these proangiogenic factors in tumor progression [97].

In melanoma, clinical progression appears to result from a complex interplay of genetic, immunological, inflammatory, and angiogenic factors. The MICA‐129 Val/Val genotype may promote increased sMICA release, reducing NK and CD8^+^ T‐cell activation and facilitating tumor immune evasion. Systemic inflammation, mediated by TNF‐α, IL‐6, and IL‐8, contributes to local immunosuppression and therapy resistance, while angiogenesis, regulated by VEGF, supports tumor growth and dissemination. In primary melanoma, immune activity is relatively preserved and angiogenesis is moderate, whereas in metastatic melanomas, angiogenesis is reactivated, immune evasion is enhanced, and the tumor microenvironment becomes proinflammatory, reflecting greater aggressiveness and a poorer prognosis. The integration of these markers provides insight into the complexity of the tumor microenvironment and may guide future therapeutic strategies.

## 5. Conclusions

The MICA‐129 Val/Val polymorphism has functional relevance in solid tumors, modulating NKG2D signaling and the activation of NK and CD8^+^ T cells, which may contribute to increased immune susceptibility and tumor immune evasion in melanoma. Disease progression is associated with the interplay of genetic, inflammatory, and angiogenic factors, as evidenced by increased IL‐6 and IL‐8 in primary melanoma and elevated TNF‐α and VEGF in metastatic cases. The integrated analysis of these markers enhances the understanding of the complexity of the tumor microenvironment and provides insights for future investigations into prognostic biomarkers and therapeutic strategies in melanoma.

## Author Contributions

**Bruna Somilio da Fonseca**: conceptualization, methodology, investigation, data curation, analysis, validation, resources, writing – original draft, writing – review and editing. **Bruna D. L. Fragelli**: methodology, investigation, data curation, analysis, validation, writing – original draft, writing – review and editing. **Joice M. A. Rodolpho**, **Krissia F. Godoy**, **Camila F. B. Castro**, **Erick C. Casteli**, **Izabela A. Lopes, and Lucas R. Azevedo**: methodology, investigation, data curation, analysis, validation, writing – review and editing. **Luciana Benevides**: methodology, writing – review and editing. **Fernanda F. Anibal**: supervision, funding acquisition, writing – review and editing.

## Funding

This research was supported by the Conselho Nacional de Desenvolvimento Científico e Tecnológico (Grants 314770/2025‐9 and 304154/2022‐9) and the Petrobras (Grant 2022/00220‐0).

## Conflicts of Interest

The authors declare no conflicts of interest.

## Supporting Information

Additional supporting information can be found online in the Supporting Information section.

## Supporting information


**Supporting Information 1** Form of free and informed consent.

**Supporting Information 2** FACT‐M questionnaire.

**Supporting Information 3** Table S1: Identified alleles and amino acids.

## Data Availability

The data that support the findings of this study are available upon request from the corresponding author. The data are not publicly available due to privacy or ethical restrictions.
