# Supplementary material for: Immunogenetic Signature of the MICA‐129 Val/Val Genotype Associated With Metastasis in Melanoma
Source: J Immunol Res. 2026 Nov 1;2026:2883849. doi: 10.1155/jimr/2883849 (PMC13725211; doi:10.1155/jimr/2883849)
Supplement: Supplementary file 1 [file JIMR-2026-2883849-s001.docx]

**Immunogenetic signature of the MICA-129 Val/Val genotype associated with**

**metastasis in melanoma**

Bruna Somilio da Fonseca^1,3*^, Bruna D. L. Fragelli^1^, Joice M. A. Rodolpho^1^, Krissia F. Godoy^1^, Camila F. B. Castro^2^, Erick C. Casteli^2^, Lucas R. Azevedo^3^, Izabela A. Lopes^4^, Luciana Benevides^4^ and Fernanda F. Anibal^1*^

^1^ Laboratory of Inflammation and Infectious Diseases, Federal University of São Carlos (UFSCar), 13565-908, São Carlos, SP, Brazil

^2^ Molecular Genetics and Bioinformatics Laboratory–Experimental Research Unit, School of Medicine, São Paulo State University (UNESP) Botucatu, 18618-687, Botucatu, SP, Brazil

^3^ Padre Albino University Centre – UNIFIPA, 15809-144, Catanduva, Brazil

^4^ Oswaldo Cruz Foundation (Fiocruz), Fiocruz Ribeirão Preto, 14049-900, Ribeirão Preto, SP, Brazil

**Below is a list of statements that other people with your condition have said are important. Please circle or tick one number per line to indicate your response, referring to the past 7 days.**

|  | **PHYSICAL WELL-BEING** | **Not at all** | | **A little** | **Medium** | **Quite a bit** | **Very** |
| --- | --- | --- | --- | --- | --- | --- | --- |
|  |  |  |  |  |  |  |  |
| GP1 | I feel tired | 0 | | 1 | 2 | 3 | 4 |
| GP2 | I feel nauseous | 0 | | 1 | 2 | 3 | 4 |
| GP3 | Because of my condition, I find it difficult to meet my family’s needs | 0 | | 1 | 2 | 3 | 4 |
| GP4 | I am in pain | 0 | | 1 | 2 | 3 | 4 |
| GP5 | I am bothered by the side effects of the treatment | 0 | | 1 | 2 | 3 | 4 |
| GP6 | I feel unwell | 0 | | 1 | 2 | 3 | 4 |
| GP7 | I feel forced to stay in bed | 0 | | 1 | 2 | 3 | 4 |
|  | | | | | | | |
|  | **SOCIAL/FAMILY WELFARE** | **Not at all** | | **A little** | **Medium** | **Quite a bit** | **Very** |
|  |  |  |  |  |  |  |  |
| GS1 | I feel close to my friends | 0 | | 1 | 2 | 3 | 4 |
| GS2 | I receive emotional support from my family | 0 | | 1 | 2 | 3 | 4 |
| GS3 | I receive support from my friends | 0 | | 1 | 2 | 3 | 4 |
| GS4 | My family has accepted my diagnosis/disease | 0 | | 1 | 2 | 3 | 4 |
| GS5 | I am satisfied with how my family communicates about my disease | 0 | | 1 | 2 | 3 | 4 |
| GS6 | I feel close to my partner (or the person who is my main source of support) | 0 | | 1 | 2 | 3 | 4 |
| Q1 | *Regardless of your current level of sexual activity, please answer the following question. If you would prefer not to answer it, please tick this box and move on to the next section* | |  | | | | |
| GS7 | I am happy with my sex life | | 0 | 1 | 2 | 3 | 4 |

**Please circle or tick one number per line to indicate your answer, referring to the last 7 days.**

|  | **EMOTIONAL WELL-BEING** | **Not at all** | **A little** | **Medium** | **Quite a bit** | **Very** |
| --- | --- | --- | --- | --- | --- | --- |
|  |  |  |  |  |  |  |
| GE1 | I feel sad | 0 | 1 | 2 | 3 | 4 |
| GE2 | I am satisfied with how I am coping with my disease | 0 | 1 | 2 | 3 | 4 |
| GE3 | I am losing hope in the fight against my disease | 0 | 1 | 2 | 3 | 4 |
| GE4 | I feel anxious | 0 | 1 | 2 | 3 | 4 |
| GE5 | I am afraid of dying | 0 | 1 | 2 | 3 | 4 |
| GE6 | I am worried that my condition will get worse | 0 | 1 | 2 | 3 | 4 |
|  | | | | | | |
|  | **FUNCTIONAL WELL-BEING** | **Not at all** | **A little** | **Medium** | **Quite a bit** | **Very** |
|  |  |  |  |  |  |  |
| GF1 | I am able to work (including working from home) | 0 | 1 | 2 | 3 | 4 |
| GF2 | My work (including working from home) is rewarding | 0 | 1 | 2 | 3 | 4 |
| GF3 | I am able to enjoy life | 0 | 1 | 2 | 3 | 4 |
| GF4 | I have come to terms with my disease | 0 | 1 | 2 | 3 | 4 |
| GF5 | I am sleeping well | 0 | 1 | 2 | 3 | 4 |
| GF6 | I am enjoying my leisure activities | 0 | 1 | 2 | 3 | 4 |
| GF7 | I am satisfied with my quality of life at the moment | 0 | 1 | 2 | 3 | 4 |

**Please circle or tick one number per line to indicate your answer, referring to the last 7 days.**

|  | **ADDITIONAL CONCERNS** | **Not at all** | **A little** | **Medium** | **Quite a bit** | **Very** |
| --- | --- | --- | --- | --- | --- | --- |
|  |  |  |  |  |  |  |
| M1 | I feel pain at the site of the injury or surgery | 0 | 1 | 2 | 3 | 4 |
| M2 | I have noticed new changes in my skin (lumps, raised areas or colour) | 0 | 1 | 2 | 3 | 4 |
| M3 | I am concerned about the appearance of my surgical scars | 0 | 1 | 2 | 3 | 4 |
| B1 | I have been feeling short of breath | 0 | 1 | 2 | 3 | 4 |
| ITU4 | I need to limit my physical activity because of my condition | 0 | 1 | 2 | 3 | 4 |
| An10 | I have headaches | 0 | 1 | 2 | 3 | 4 |
| Hep3 | I have had a fever (episodes of high body temperature) | 0 | 1 | 2 | 3 | 4 |
| C1 | I have swelling or cramps in my stomach area | 0 | 1 | 2 | 3 | 4 |
| C6 | My appetite is fine | 0 | 1 | 2 | 3 | 4 |
| M5 | I feel pain and discomfort in my bones | 0 | 1 | 2 | 3 | 4 |
| M6 | I have noticed blood in my stools | 0 | 1 | 2 | 3 | 4 |
| ITU3 | I need to limit my social activities because of my condition | 0 | 1 | 2 | 3 | 4 |
| MS8 | I feel overwhelmed by my condition | 0 | 1 | 2 | 3 | 4 |
| M8 | I isolate myself from others because of my condition | 0 | 1 | 2 | 3 | 4 |
| M9 | I have difficulty thinking clearly (remembering and concentrating) | 0 | 1 | 2 | 3 | 4 |
| HI7 | I feel tired | 0 | 1 | 2 | 3 | 4 |

**Please circle or tick one number per line to indicate your answer, referring to the last 7 days.**

|  | ***At the site of the surgical procedure for the injury:*** | **Not at all** | **A little** | **Medium** | **Quite a bit** | **Very** |
| --- | --- | --- | --- | --- | --- | --- |
|  |  |  |  |  |  |  |
| M10 | I have swelling at the site of my injury | 0 | 1 | 2 | 3 | 4 |
| M11 | I have swelling as a result of the surgery | 0 | 1 | 2 | 3 | 4 |
| M12 | I am bothered by the amount of swelling | 0 | 1 | 2 | 3 | 4 |
| M13 | Moving the swollen area is painful | 0 | 1 | 2 | 3 | 4 |
| M14 | The swelling prevents me from doing the things I want to do | 0 | 1 | 2 | 3 | 4 |
| M15 | The swelling prevents me from wearing the clothes or shoes I want to wear | 0 | 1 | 2 | 3 | 4 |
| M16 | I feel numbness at the site of the surgery | 0 | 1 | 2 | 3 | 4 |
| M17 | I have a good range of motion in my arm or leg | 0 | 1 | 2 | 3 | 4 |
