# Supplementary material for: Immunogenetic Signature of the MICA‐129 Val/Val Genotype Associated With Metastasis in Melanoma
Source: J Immunol Res. 2026 Nov 1;2026:2883849. doi: 10.1155/jimr/2883849 (PMC13725211; doi:10.1155/jimr/2883849)
Supplement: Supplementary file 2 [file JIMR-2026-2883849-s002.docx]

**Immunogenetic signature of the MICA-129 Val/Val genotype associated with metastasis in melanoma**

Bruna Somilio da Fonseca^1,3*^, Bruna D. L. Fragelli^1^, Joice M. A. Rodolpho^1^, Krissia F. Godoy^1^, Camila F. B. Castro^2^, Erick C. Casteli^2^, Lucas R. Azevedo^3^, Izabela A. Lopes^4^, Luciana Benevides^4^ and Fernanda F. Anibal^1*^

^1^ Laboratory of Inflammation and Infectious Diseases, Federal University of São Carlos (UFSCar), 13565-908, São Carlos, SP, Brazil

^2^ Molecular Genetics and Bioinformatics Laboratory–Experimental Research Unit, School of Medicine, São Paulo State University (UNESP) Botucatu, 18618-687, Botucatu, SP, Brazil

^3^ Padre Albino University Centre – UNIFIPA, 15809-144, Catanduva, Brazil

^4^ Oswaldo Cruz Foundation (Fiocruz), Fiocruz Ribeirão Preto, 14049-900, Ribeirão Preto, SP, Brazil

**FORM OF FREE AND INFORMED CONSENT**

Research project title: “Identification of polymorphisms in the VEGF and PDGF-BB genes in patients with melanoma”

Principal investigator: Bruna Somilio da Fonseca

Supervisor: Prof. Dr. Fernanda de Freitas Anibal

**Participant’s name:**

**Date of birth:**

You are invited to take part in a research project entitled:

“Identification of polymorphisms in the VEGF and PDGF-BB genes in patients with melanoma”, led by researchers Bruna Somilio da Fonseca and Prof. Dr. Fernanda de Freitas Anibal.

Please read this form carefully and ask if you have any questions. If you are satisfied with the information provided and agree to take part in the study, Please sign at the end of this document, in duplicate: one copy for you and one for the principal investigator.

It is important to note that your participation is voluntary. You are fully entitled to refuse or withdraw from the study at any time, without this causing you any disadvantage. There is no cost to you for taking part, nor will there be any financial benefit.

**1. Objective of the Study**

This study aims to identify polymorphisms in the VEGF and PDGF-BB genes in patients with melanoma. The research will be conducted at the Catanduva School of Medicine – UNIFIPA and at the Emílio Carlos Teaching Hospital, in partnership with the Laboratory of Inflammation and Infectious Diseases (LIDI) at the Federal University of São Carlos (UFSCar), and the Neopelle Clinic.

**2. Participation Procedure**

Participation will consist of blood collection by venepuncture, performed by a duly trained team. The first collection will take place at Clínica Neopelle. It should be noted that a second collection may be required, which will be communicated to the participant in advance.

**3. Questionnaire Administration**

An adapted FACT-M questionnaire will be administered in order to assess the quality of life of patients diagnosed with cutaneous carcinoma and melanoma.

**4. Risks Involved**

During blood collection, bruising at the puncture site or mild discomfort at the moment of needle insertion may occur. Such symptoms will be minimised by qualified professionals, who will follow standard blood collection protocols.

**5. Expected Benefits**

Among the benefits of participation is the contribution to the advancement of science, with the potential discovery of therapeutic tools. In the future, participants may benefit directly or indirectly from the results of the research.

The estimated duration of participation is up to 30 minutes, including blood sample collection, reading, clarification of any questions, and signing of this consent form.

**6. Financial Aspects**

There will be no payment to, nor any charges for, the volunteers. Should any expenses arise directly from participation, these will be reimbursed by the researchers.

**7. Guarantee of Rights**

In the event of harm demonstrably caused by participation in the research, volunteers may seek compensation, as provided for in the Civil Code (Law N^o^. 10,406/2002) and in Resolutions N^o^. 466/2012 and 510/2016 of the National Health Council.

**8. Privacy and Confidentiality**

Participants’ names will be kept confidential, ensuring their privacy. All participants will have full access to the information and may ask questions before, during, and after their participation.

**9. Use of Data**

The data collected will be used exclusively for this research, and the results may be published while preserving the identity of the participants.

**Queries and Contacts:**
Bruna Somilio da Fonseca – Principal Investigator
📞 Telephone: (17) 98115-3296
📧 Email: brunasomilio@msn.com

**National Research Ethics Commission – CONEP**
📞 Telephone: (61) 3315-5877
📧 Email: conep@saude.gov.br

Catanduva, of	2025.

Participant’s Signature

Assinatura do pesquisador
