# Supplementary material for: Immunogenetic Signature of the MICA‐129 Val/Val Genotype Associated With Metastasis in Melanoma
Source: J Immunol Res. 2026 Nov 1;2026:2883849. doi: 10.1155/jimr/2883849 (PMC13725211; doi:10.1155/jimr/2883849)
Supplement: Supplementary file 3 [file JIMR-2026-2883849-s003.docx]

**Immunogenetic signature of the MICA-129 Val/Val genotype associated with metastasis in melanoma**

Bruna Somilio da Fonseca^1,3*^, Bruna D. L. Fragelli^1^, Joice M. A. Rodolpho^1^, Krissia F. Godoy^1^, Camila F. B. Castro^2^, Erick C. Casteli^2^, Lucas R. Azevedo^3^, Izabela A. Lopes^4^, Luciana Benevides^4^ and Fernanda F. Anibal^1*^

^1^ Laboratory of Inflammation and Infectious Diseases, Federal University of São Carlos (UFSCar), 13565-908, São Carlos, SP, Brazil

^2^ Molecular Genetics and Bioinformatics Laboratory–Experimental Research Unit, School of Medicine, São Paulo State University (UNESP) Botucatu, 18618-687, Botucatu, SP, Brazil

^3^ Padre Albino University Centre – UNIFIPA, 15809-144, Catanduva, Brazil

^4^ Oswaldo Cruz Foundation (Fiocruz), Fiocruz Ribeirão Preto, 14049-900, Ribeirão Preto, SP, Brazil

**Table S1 –** Identified alleles and their corresponding amino acids

| Group | Sample | Allele | Amino Acid |
| --- | --- | --- | --- |
| M+M | 1 | A/A | Met/Met |
| M+M | 5 | G/A | Val/Val |
| M+M | 8 | G/A | Val/Val |
| M+M | 14 | G/A | Val/Val |
| M+M | 18 | G/A | Val/Val |
| M+M | 20 | A/A | Met/Met |
| M | 4 | G/A | Val/Met |
| M | 7 | G/A | Val/Met |
| M | 10 | G/A | Val/Met |
| M | 11 | G/G | Val/Val |
| M | 13 | G/G | Val/Val |
| M | 16 | G/G | Val/Val |
| M | 19 | G/A | Val/Met |
| M | 21 | G/A | Val/Met |
| M | 22 | G/A | Val/Met |
| M | 23 | G/A | Val/Met |
| M | 24 | G/A | Val/Met |
| M | 25 | G/G | Val/Val |
| M | 27 | G/G | Val/Val |
| M | 29 | G/G | Val/Val |
| M | 30 | G/A | Val/Met |
| M | 31 | G/A | Val/Met |
| M | 33 | G/A | Val/Met |
| M | 35 | G/G | Val/Val |
| BCC | 2 | G/A | Val/Met |
| BCC | 3 | G/A | Val/Met |
| BCC | 6 | G/A | Val/Met |
| BCC | 9 | G/A | Val/Met |
| BCC | 12 | A/A | Met/Met |
| BCC | 15 | G/A | Val/Met |
| BCC | 17 | G/A | Val/Met |
| BCC | 26 | G/A | Val/Met |
| BCC | 28 | A/A | Met/Met |
| BCC | 32 | G/A | Val/Met |
| BCC | 34 | G/A | Val/Met |
